# Constitutional multicenter bank linked to Sasang constitutional phenotypic data

**DOI:** 10.1186/s12906-015-0553-3

**Published:** 2015-03-10

**Authors:** Hee-Jeong Jin, Younghwa Baek, Ho-Seok Kim, Jonghyang Ryu, Siwoo Lee

**Affiliations:** KM Health Technology Research Group, Korea Institute of Oriental Medicine, 461-24, Jeonmin-dong, Yuseong-gu, Daejeon, 305-811 South Korea

**Keywords:** Biobank, Sasang Constitutional Medicine, Sasang Constitution

## Abstract

**Background:**

Biobanks are more important in medical area because they can give researchers data for demonstrating and validating their research. In this study, we developed a biobank called the Korea Constitutional Multicenter Bank (KCMB) based on Sasang Constitutional Medicine (SCM). The aim of the KCMB was a foundation to providing the scientific basis of SCM.

**Methods:**

The KCMB has been constructed since 2006 in 24 Korean medical clinics with collection of questionnaire data, physical measurements and biological information comprised the results from blood test and DNA analyses. All participants were prescribed Sasang Constitution (SC)-specific herbal remedies for the treatment, and showed improvement of original symptoms as confirmed by Korean medicine doctor. Collected data went through de-identification process using the electronic case report form system. For calculation of several SC type specific tendencies, we used the direct standardization and Chi-square tests.

**Results:**

The KCMB collected clinical information from 3,711 study participants (1,353 men and 2,358 women) aged more than 10 years. The mean age (± standard deviation) was 47.1 (±16.6) and 47.7 (±15.8) years for men and women respectively. After applying the direct standardization, the estimated constitutional distributions for the SC types were as follows: 39.2% for Tae-eumin(TE), 27.1% for Soeumin(SE), 33.7% for Soyangyin(SY), and non-zero but below 0.1% for Taeyangyin(TY). The estimated distribution of TE was about 10% less, while that of SY and SE were slightly more than the distribution reported by Jema Lee established the SCM. Based on the participants’ medical history within the KCMB, each SC type had notably different frequencies for some diseases such as hypertension, diabetes, hyperlipidemia, stroke, and obesity (P < 0.001).

**Conclusions:**

The KCMB may serve to verify and validate SCM theories and practices. It may also provide new insights into SCM mechanisms. The results from many studies using the KCMB data are of great importance and value for making decisions in healthcare policy and developing novel therapies.

## Background

Many researches have studied methods and theories for preventing and diagnosing diseases, and identifying individual differences for personalized medicine. With these efforts, significant progresses in clinical medicine have been made until now. Biobanks played a vital role here, because they provide the biological and clinical information required for research to identify useful biomarkers [1].

The UK biobank was established and hosted by the University of Manchester and supported by the National Health Service (NHS) [2,3]. It aims to build a major resource that can support a diverse range of research intended to improve the prevention, diagnosis, and treatment of illness and the promotion of health throughout the society. The China Kadoorie biobank (CKB) is set up to investigate the main genetic and environmental causes of common chronic diseases in the Chinese population [4,5]. In the CKB, 512,891 adults aged 30–79 years were recruited, of which 41% were men, 56% were from rural areas, and mean age was 52 years [4]. The Framingham Heart Study was established under the direction of the National Heart, Lung, and Blood Institute (NHLBI) in 1948 [6,7], and until 2012, 2,473 articles were published in peer-reviewed medical journals based on Framingham Heart Study data. These efforts enabled the basis of the western medicine to have been accumulated. On the other hand, it is only recently that these biobanks have gained interest in oriental medicine.

In Korea, Sasang Constitutional Medicine (SCM) is one of the Korea’s unique traditional medicines [8-11]. SCM classifies people into 4 sasang constitution (SC) types, namely Taeyangyin (TY), Tae-eumin (TE), Soyangyin (SY), and Soeumin (SE). These classifications are based on the characteristics of an individual’s physiology, psychology, and physical attributes. The SC type of a person is thought to determine his/her response to different herbal remedies [8]. The medicinal herbs used in SCM are similar to those used in traditional China medicine (TCM), but the basic principles underlying the choice of treatment and prescription of these remedies are completely different [12]. In SCM, the SC type of the patient is the primary consideration for selecting the medicinal herbs and formulae for treatment. In contrast, TCM medicinal herbs are classified according to the therapeutic effects of the herb itself, namely, dispersive quality, Yin tonifying quality and so forth [13].

Several recent studies have reported correlations between the SC types and an individual’s characteristics [14-19]. It is thought that knowledge regarding an individual’s SC type can be used to better treat disease and improve the quality of health care. Most of these studies have focused on finding different features and their biological mechanism based on SC types [17,18,20-26]. One of the important things for these researches is that they should require biological and clinical information based on SCM.

In this paper, we introduce the Korea Constitutional Multicenter Bank (KCMB) based on the previous SCM researches [27-34], and show several distinctive tendencies of diseases with respect to SC types using the biological and clinical data from this biobank.

## Methods

### Data source and subjects

**Figure 1:**
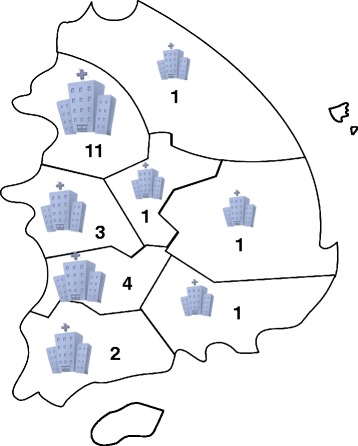
**Distribution of hospitals that participated in the KCMS.**

The Korea Constitutional Multicenter Study (KCMS) is an ongoing project designed to establish a database for SCM [35]. Currently, 3,711 participants are enrolled from 24 Korean medical clinics (KMCs) since August 2012. The KCMS was approved by the Institutional Review Board at the Korea Institution of Oriental Medicine (KIOM) (I-0910/02-001). The Korea Constitutional Multicenter Bank (KCMB) is a biobank linked to phenotypic data from KCMS. Figure 1 shows the distribution of hospitals contributing to this study. The hospitals are distributed fairly evenly amongst the different regions of Korea, and the study population represents the entire population of Korea. Figure 1 also shows a concentration of 11 hospitals in a population-dense area where approximately 20% of the Korean population resides.

All participants in the KMCS have had documented responses to herbal medicine as confirmed by Korean medicine doctor (KMD). In order to diagnose the SC type, each participant was prescribed a SC-specific herbal remedy for the treatment of their most prominent physical discomfort [21]. After taking the medicine for 30 days or more, improvement of original symptoms and occurrence of adverse effects were recorded. The SC types were determined only for participants who had an obvious improvement in their chief complaints without experiencing any adverse effects such as indigestion, stomachache, and evacuation troubles. Every hospital recruited participants within their respective patient population. To ensure the accuracy of the diagnoses, practitioners who took part in this study were restricted to those who had more than 5 years of experience in clinical practice. A more detailed description of the verification methodology and procedure of SC types that we used have previously been reported in [35].

### The flow of collecting and validation of data in KCMS

We collected and recorded various clinical and biological data. Clinical information was obtained using the Case Report Form (CRF) which is a self-reported questionnaire developed by KIOM to be used for standardization of SCM. Every question on the CRF was designed by SCM experts with reference to Jema Lee’s book Donguisusebowon [9]. The CRF consists of 7 parts: (1) general information, (2) external appearance, (3) somatotype, (4) personality, (5) general health condition, (6) symptoms, and (7) reaction to medication. The general information consists of personal information, including gender, age, and marital status. Some sections in the reaction-to-medication part are subjective opinions to be written by the KMD.

The biological information comprised the results from blood test and DNA analyses. The aim of these data was to find genetic and biological characteristics of different SC types. Genomic DNA was isolated from the peripheral blood of participants and was genotyped using the Affymetrix Genome Wide Human SNP array 5.0 [21].

**Figure 2:**
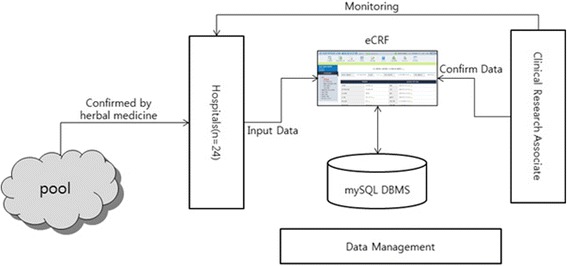
**Flow chart of collecting constitutional data in KCMB.**

Figure 2 shows the flow chart of the process that the KCMB employed to collect data. The information on every questionnaire was recorded using the KCMB DBMS system by means of an electronic CRF (eCRF) that was inputted by a designated researcher at each hospital. The Clinical Research Associate (CRA) checked and confirmed the accuracy of the data. A data-management expert performed quality-control checks from time to time to ensure accuracy of the recorded data.

### Electronic case report form system

The use of eCRF to gather data in clinical and biological researches have grown to progressively replace paper-based CRFs [36]. Many reports show that efficiency for data collection, reporting, query resolution, and validation can be improved by replacing paper-based CRF with electronic ones (eCRF) [37-39]. Several requirements for development of the eCRF is recommended by the US Food and drug administration (FDA) [40] and Society for Clinical Data Management [41]. In Korea, the requirements for the eCRF was ordained by Korea FDA [42].

**Figure 3:**
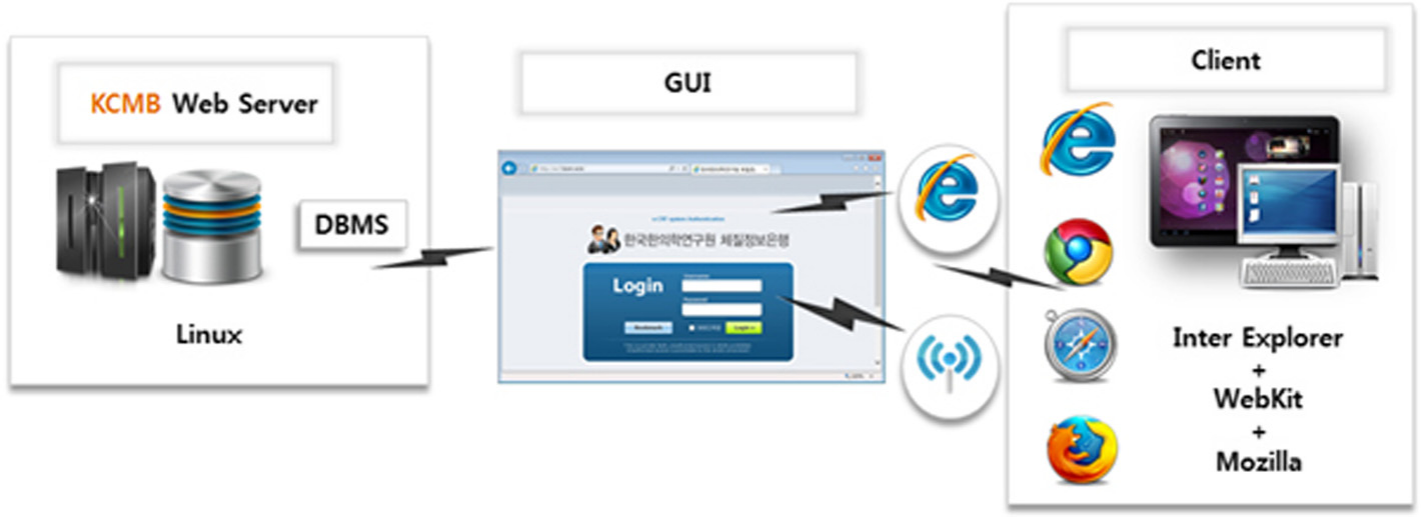
**Structure of the eCRF for KCMB: all-purpose client–server architecture is useful and a relational DBMS was applied for clinical and biological data storage.** The client can communicate with the eCRF on-line via Internet.

In our study, we established the eCRF which has been developed in compliance with the guidelines [42]. Figure 3 shows the structure of our eCRF system. Our eCRF system supported various client operating systems and Internet browsers, and used a relational database management system to collect and manage clinical and biological data.

### Standardization method

We used the direct standardization method to estimate the distribution of SCM [43-45]. There are several methods that can be used for the estimation of standardization rates [14,46], and many researchers have used direct and indirect standardization methods [43,47-49]. Standardization allows for comparisons between groups even though there are variations in the number of individuals in each group. Sub-groups are usually defined by age or age and gender. For standardization, the weighted sum (or weighted average) of the sub-group–specific rates in the study population is calculated. Direct standardization calculates a weighted average of the region’s age-specific mortality rates where the weights represent the age-specific sizes of the standard population. Indirect standardization uses age-specific mortality rates from the standard population to derive expected distribution in the region’s population.

In most circumstances a single easy-to-interpret ratio can be obtained from the direct standardized rate by dividing the expected number in the standard population by the observed number in standard population over the same time period [50]. However, obtaining an easy-to-interpret ratio may be difficult when artificial populations are used because no observations are available. It may be necessary to use standardized rates in these circumstances, and thus standardized rates are used for the purposes of the study.

## Results

### General characters

Collected resources are consisted of two parts, clinical and biological information in KCMB (Table 1). The 3,711 clinical information and 3,691 biological information are collected.

**Table 1:** **The status of collected clinical and biological information**

**Category**	**Number of cases**
Clinical information	
Questionnaire	3,711
Face picture	3,689
Body shape	3,610
Voice record	3,229
Biological information	
DNA	3,691
Serum	3,689
Blood exam	3,628
EBV	2,886
SNP	1,104

EBV: Epstein–Barr virus, SNP: Affymetrix Genome Wide Human SNP array 5.0.

Data from 3,711 participants, 1,353 (36.46%) men and 2,358 (63.54%) women, were collected in the KCMS. The mean age (± standard deviation) was 47.1 (±16.6) and 47.7 (±15.8) years for men and women respectively; the ages were not statistically different (P > 0.05). Table 2 shows detailed characteristics of the study population, and Table 3 shows the general characteristic of participants according to SC types. The distribution of the population according to the SC types for men was as follows: TE 585 (43.2%), SE 314 (23.2%), SY 430 (31.8%), and TY 24 (1.8%). The distribution for women was as follows: TE 883 (37.4%), SE 640 (27.1%), SY 783 (33.2%), and TY 52 (2.2%).

**Table 2:** **The general characteristics according to gender of participants**

	**Total**	**Male**	**Female**	**P-value**
	**(n = 3,711)**	**(n = 1,353)**	**(n = 2,358)**	
Age (years)	47.5 ± 16.1	47.1 ± 16.6	47.7 ± 15.8	0.332
Height (cm)	161.8 ± 8.6	169.4 ± 7.0	157.4 ± 6.1	0.000
Weight (kg)	61.2 ± 11.3	68.6 ± 11.4	57.0 ± 8.8	0.000
BMI (kg/m^2^)	23.3 ± 3.3	23.8 ± 3.2	23 ± 3.4	0.000
SBP (mmHg)	119.3 ± 15.7	122.4 ± 15.1	117.6 ± 15.7	0.000
DBP (mmHg)	76.7 ± 11.2	78.8 ± 11.2	75.5 ± 11.1	0.000
Marital status				
Marriage	2702(73.6)	1002(74.9)	1700(72.8)	0.081
Unmarried	971(26.4)	335(25.1)	636(27.2)	
Occupation				
White-color	1317(36.3)	619(46.9)	698(30.2)	0.000
Blue-color	534(14.7)	301(22.8)	233(10.1)	
Other	1782(49.1)	399(30.3)	1383(59.8)	
Education (years)				
<6	735(20.1)	177(13.3)	558(24)	0.000
6-12	1431(39.1)	544(41)	887(38.1)	
>12	1490(40.8)	607(45.7)	883(37.9)	

BMI, body mass index; SBP, systolic blood pressure; DBP, diastolic blood pressure.

Means of variables were not significantly different between men and women, P > 0.05. Distribution of participants was significantly different among variable categories and between gender.

**Table 3:** **The general characteristics by Sasang constitution type**

	**Male**				**Female**			
	**TE**	**SE**	**SY**	**TY**	**TE**	**SE**	**SY**	**TY**
Number	585(43.2)	314(23.2)	430(31.8)	24(1.8)	883(37.4)	640(27.1)	783(33.2)	52(2.2)
Age (years)	48.2 ± 16.6	42 ± 16.8	49.6 ± 15.6	43.1 ± 18.1	50 ± 16.4	45.7 ± 15.9	46.8 ± 15.1	44.2 ± 12.2
Height (cm)	169.7 ± 7.2	169.2 ± 7.6	169 ± 6.4	171.1 ± 5.9	157.6 ± 6	158 ± 5.9	156.8 ± 6.2	158.6 ± 5.9
Weight (kg)	73.6 ± 11.7	62 ± 9.4	66.9 ± 9.3	62.9 ± 10.4	62.2 ± 9	52.5 ± 6.7	55.1 ± 7.2	53.4 ± 7.9
BMI (kg/m^2^)	25.5 ± 3.0	21.6 ± 2.6	23.4 ± 2.7	21.4 ± 2.9	25.1 ± 3.3	21 ± 2.6	22.4 ± 2.8	21.2 ± 3.0
SBP (mmHg)	125.8 ± 14.5	117.4 ± 15.1	121.6 ± 15	118.1 ± 14	121 ± 16.3	115.1 ± 15.2	116 ± 14.9	113 ± 12.5
DBP (mmHg)	80.6 ± 10.6	75.9 ± 11.6	78.7 ± 11.1	74.3 ± 9.5	77.6 ± 11.2	73.6 ± 10.8	74.7 ± 10.9	73.9 ± 9.0
Marital status								
Marriage	440(76.4)	208(66.7)	337(79.3)	17(70.8)	638(73.5)	448(70.4)	570(73.1)	44(84.6)
Unmarried	136(23.6)	104(33.3)	88(20.7)	7(29.2)	230(26.5)	188(29.6)	210(26.9)	8(15.4)
Occupation								
White-color	262(45.8)	153(49.8)	188(45.2)	16(66.7)	224(26.1)	192(30.3)	265(34.4)	17(33.3)
Blue-color	134(23.4)	50(16.3)	115(27.6)	2(8.3)	97(11.3)	53(8.4)	78(10.1)	5(9.8)
Other	176(30.8)	104(33.9)	113(27.2)	6(25)	538(62.6)	389(61.4)	427(55.5)	29(56.9)
Education (years)								
<6	80(14)	38(12.3)	57(13.5)	2(8.3)	261(30.1)	129(20.4)	161(20.7)	7(13.5)
6-12	230(40.1)	125(40.5)	181(42.9)	8(33.4)	353(40.8)	227(36)	290(37.2)	17(32.7)
>12	263(45.9)	146(47.2)	184(43.6)	14(58.3)	252(29.1)	275(43.6)	328(42.1)	28(53.8)

BMI, body mass index; SBP, systolic blood pressure; DBP, diastolic blood pressure; TE, Tae-eumin; SE, Soeumin; SY, Soyangin; TY, Taeyangyin.

### Frequency of diseases by SC

Participants’ medical history was included in the KCMB. Table 4 shows the frequency of various diseases, including hypertension, diabetes, hyperlipidemia, stroke, and obesity for each of the SC types. It is notable that the frequencies of these diseases are significantly different between different SC types (P < 0.001, see Table 4 for detail).

**Table 4:** **Frequency of diseases by Sasang constitution type**

	**TE**	**SE**	**SY**	**P-value**
Total				
Hypertension	466(31.7%)	150(15.7%)	259(21.4)	0.000
Diabetes	164(11.2%)	44(4.6%)	108(8.9)	0.000
Hyperlipidemia	248(16.9%)	93(9.7%)	132(10.9)	0.000
Stroke	230(16.0%)	82(8.7%)	136(11.4)	0.000
Obesity	317(25.4%)	31(3.7%)	93(8.7)	0.000
Male				
Hypertension	196(33.5%)	47(15%)	132(30.7)	0.000
Diabetes	68(11.6%)	16(5.1%)	54(12.6)	0.001
Hyperlipidemia	115(19.7%)	31(9.9%)	58(13.5)	0.001
Stroke	118(20.3)	35(11.4)	90(21)	0.001
Obesity	102(20.9)	9(3.2)	29(7.9)	0.000
Female				
Hypertension	270(30.6)	103(16.1)	127(16.2)	0.000
Diabetes	96(10.9)	28(4.4)	54(6.9)	0.000
Hyperlipidemia	133(15.1)	62(9.7)	74(9.5)	0.001
Stroke	112(13)	47(7.4)	46(6)	0.000
Obesity	215(28.3)	22(3.9)	64(9.2)	0.000

### Constitution frequency

The results from direct and indirect standardization methods are expected to differ slightly. We used the direct standardization method because our study included the standard population from the “2010 Korean Population and Housing Census” [51], which encompasses all Koreans and foreigners residing in the territory of the Republic of Korea, as of 0 o’clock on November 1, 2010. This was the 18th population census and 10th housing census. This census was a statistical survey intended to ascertain the demographics of the entire population as well as the number, structure, distribution, and characteristics of households and housings in the Republic of Korea.

After applying the direct standardization method, the estimated constitutional distribution amongst all participants was as follows: TE 39.2%, SE 27.1%, and SY 33.7%. Because of its small sample size, we excluded the 76 TY-type subjects completely. The distribution rates for men and women with the TE, SE, and SY constitutions were 43.5%, 24.1%, and 32.4% and 37.3%, 28.7%, and 34.0%, respectively. The rate for TE amongst men was slightly more than that for women, and the rates of SE and SY for men were slightly lesser than that for women (Table 5).

**Table 5:** **The estimated distribution of Sasang constitution types according to data from the Korea Constitutional Multicenter Study**

	**Total**			**Male**			**Female**		
	**TE**	**SE**	**SY**	**TE**	**SE**	**SY**	**TE**	**SE**	**SY**
Collected Participants									
20 ~ 29	133	127	113	50	39	35	83	88	78
30 ~ 39	208	197	220	90	58	61	118	139	159
40 ~ 49	295	206	279	115	67	97	180	139	182
50 ~ 59	325	186	268	138	56	94	187	130	174
60 ~ 69	277	104	192	102	35	80	175	69	112
70 ~ 79	143	59	85	46	16	42	97	43	43
Total	1381	879	1157	541	271	409	840	608	748
(%)	40.4	25.7	33.9	44.3	22.2	33.5	38.3	27.7	34.1
Expected Participants									
20 ~ 29	2,351,343	2,245,268	1,997,758	1,382,329	1,078,217	967,630	1,055,398	1,118,976	991,819
30 ~ 39	2,594,008	2,456,825	2,743,662	1,690,893	1,089,687	1,146,050	1,097,135	1,292,388	1,478,343
40 ~ 49	3,103,090	2,166,904	2,934,787	1,696,589	988,447	1,431,036	1,468,997	1,134,392	1,485,319
50 ~ 59	2,738,856	1,567,468	2,258,502	1,556,678	631,696	1,060,346	1,262,957	877,991	1,175,158
60 ~ 69	1,930,977	724,988	1,338,439	888,517	304,883	696,876	1,034,332	407,822	661,973
70 ~ 79	1,320,573	544,852	784,956	479,293	166,711	437,616	830,469	368,146	368,146
Total	14,470,090	9,210,144	12,123,022	7,839,624	3,927,057	5,926,814	6,927,231	5,013,996	6,168,534
(%)	39.2	27.1	33.7	43.5	24.1	32.4	37.3	28.7	34.0

TE, Tae-eumin; SE, Soeumin; SY, Soyangin.

## Discussion

The KCMB has been provided various data for the study of the clinical, biological and outcome related to SCM. The KCMB may serve to verify SCM theories in these areas and may provide new insights into SCM mechanism and course.

Through participants’ medical history within the KCMB, each constitution has a different frequency based on several diseases. According to previous researches, abdominal obesity (AO), hypertension and diabetes mellitus were revealed to be associated with a specific SC type [23,52,53]. In the AO case, specifically, the TE type was associated with increased prevalence of AO compared with the SE and SY types in males and females, even after adjusting for the potential variables such as; age, BMI, and several chronic diseases [27]. In addition, the TE type were more afflicted with diabetes mellitus than the SY or SE types [52]. Also, the TE type exhibited highest prevalence of hypertension than any other SC types, and could act as an independent risk factor for hypertension [54]. These results can be seen similarly in the KCMB and are predicted by the KCMB. Through these results, the significantly different frequency of diseases based on SC types may be an important factor considered when making decisions on healthcare policies or developing new drugs, as it could reflect the results of previous SCM studies. There are currently many efforts under way aimed at finding biological understanding about SC type. However, so far there are not many biological results. In the future, if more biological resources based SCM are collected, we will have more meaningful results.

Since Jema Lee established the SCM, most of the studies related to SCM have assumed the distribution of the SC types as the standard distribution described in Donguisusebowon [9]. However, there is no statistical evidence for this assumption, and Jema Lee only briefly referred to the distribution of the SC types. In our study, we used the direct standardization method [44,45] to estimate the distribution of the SC types in Korea based on KCMS participants. Jema Lee described the SC type distribution as follows: TE 50.0%, SE 20.0%, SY 30.0%, and TY being very rare. Our results show the estimated constitutional distribution to be TE 39.2%, SE 27.1%, and SY 33.7% (Table 5). Clearly, our estimated distribution of TE is about 10% less, while that of SY and SE is slightly more than the distribution reported by Jema Lee. The difference seems to be due to several reasons. First of all, although Jema Lee described the distribution of SC several times in his books, each of these descriptions differs slightly, and there are no evidences to substantiate these distribution rates. Second, the current Korean population structure and the population structure at the time of Jema Lee’s book differ markedly. During the past century, Korea has undergone rapid social change such as the South–north division, the Korean War, and industrialization. Third, Jema Lee’s opinion on the distribution of SC types was not meant to give an absolutely accurate distribution rate, but rather an overview of knowledge that could be applied in clinics and be used for future references. It is thus necessary to accurately estimate the distribution rate of SC types in the current era.

## Conclusion

In this paper, we overview the Korea Constitutional Multicenter Bank (KCMB) and showed several tendency based on Sasang Constitutional (SC) types. Recently, increasing number of studies has reported correlations between the SC types and an individual’s characteristics. Estimation of the distribution rate of SC using the KCMB could be used to calculate the prevalence of various diseases according to SC types. We expect that more studies might be progressed for providing the scientific advancement of SCM using the KCMB with various data. The results from these studies are useful and should be considered when making decisions on healthcare policies or developing novel therapies.

## Abbreviations

TE: Tae-eumin
SE: Soeumin
SY: Soyangin
TY: Taeyangyin
